# Complement-Mediated Hemolytic Anemia Triggered by Adenovirus Infection in a Pediatric Patient

**DOI:** 10.7759/cureus.104180

**Published:** 2026-02-24

**Authors:** Gordon Fuller, Malcolm Anderson

**Affiliations:** 1 Pediatrics, Rocky Vista University College of Osteopathic Medicine, Denver, USA; 2 Pediatric Intensive Care Unit, Rocky Vista University College of Osteopathic Medicine, Denver, USA

**Keywords:** adenovirus infection, cold-reactive autoimmune hemolytic anemia, complement-mediated hemolysis, donath–landsteiner antibody, paroxysmal cold hemoglobinuria (pch)

## Abstract

Paroxysmal cold hemoglobinuria (PCH) is a rare form of complement-mediated hemolytic anemia in children. It is typically triggered by viral infections and mediated by Donath-Landsteiner antibodies, which induce intravascular hemolysis upon cold exposure. We reviewed the clinical course, laboratory findings, and management of a pediatric adenovirus-associated PCH to highlight diagnostic and therapeutic considerations. The eight-year-old previously healthy, unimmunized female patient presented with acute hemolytic anemia following a febrile viral illness. She developed jaundice and severe anemia with reticulocytosis, hyperbilirubinemia, and elevated lactate dehydrogenase (LDH). The direct antiglobulin test (DAT) was positive for complement (C3) but negative for immunoglobulin G (IgG). Blood bank evaluation revealed a positive Donath-Landsteiner antibody, confirming PCH. Treatment included normothermia, intravenous methylprednisolone, intravenous immunoglobulin (IVIG), epoetin alfa, folic acid, and empiric azithromycin. The patient’s clinical condition stabilized with gradual hematologic recovery and resolution of symptoms. This case identifies adenovirus as a potential trigger for PCH in children and emphasizes the importance of early recognition, laboratory confirmation, and supportive management.

## Introduction

Cold-reactive autoimmune hemolytic anemias (AIHAs) are rare disorders characterized by immune-mediated destruction of red blood cells through temperature-dependent antibody activation and complement activation. In pediatric populations, these conditions are most often acute and postinfectious in nature, with viral illnesses serving as common triggers [1-3]. Paroxysmal cold hemoglobinuria (PCH) represents an uncommon subtype of cold-reactive AIHA and is mediated by biphasic immunoglobulin G (IgG) autoantibodies, known as Donath-Landsteiner antibodies, which bind erythrocytes at low temperatures and initiate complement-mediated intravascular hemolysis upon rewarming [2,4].

Historically associated with syphilis, PCH is now predominantly observed in children following viral infections and typically presents with sudden-onset anemia, jaundice, hemoglobinuria, and laboratory evidence of hemolysis [2,5]. Differentiating PCH from other forms of AIHA, including cold agglutinin disease (CAD) and warm AIHA, is essential due to differences in immunopathogenesis, clinical course, and management strategies [1,3,6]. While *Mycoplasma pneumoniae* and Epstein-Barr virus are well-established precipitants, adenovirus-associated PCH has been infrequently reported in the literature [7]. A 2023 systematic review identified 230 reported cases of PCH confirmed by Donath-Landsteiner antibody testing, of which only three were associated with antecedent adenovirus infection [8].

This report describes a pediatric case of adenovirus-triggered PCH and highlights key diagnostic features, laboratory confirmation, and therapeutic considerations relevant to the management of this condition.

## Case presentation

An eight-year-old previously healthy, unimmunized female patient presented with a two-day history of low-grade fever, nausea, vomiting, decreased appetite, and headache, followed by the onset of scleral icterus and progressive jaundice. There was no associated rash, diarrhea, or weight loss, though several household sick contacts were reported. Initial evaluation revealed severe anemia with laboratory evidence of hemolysis (Table 1). Due to a rapid decline in hemoglobin and concern for acute hemolytic anemia, she was transferred to a tertiary pediatric ICU.

**Table 1:** Key laboratory findings during hospital course DAT: Direct antiglobulin test; IgG: Immunoglobulin G; INR: International normalized ratio; LDH: Lactate dehydrogenase; PCR: Polymerase chain reaction

Laboratory Parameter	Reference Range	Initial Presentation	Nadir/Peak	Recovery Phase
Hemoglobin (g/dL)	11.5–15.5	6.8	4.2	4.8
White Blood Cell Count (×10⁹/L)	4.5–13.5	15.6	15.6	12.1
Platelets (×10⁹/L)	150–450	480	480	410
Reticulocyte Count (%)	0.5–2.0	8.0	8.4	5.0
Absolute Reticulocyte Count (×10⁶/µL)	0.02–0.10	0.133	0.133	0.098
Immature Reticulocyte Fraction (%)	5–25	32.8	51.5	28.4
LDH (U/L)	110–295	~800	684	410
Total Bilirubin (mg/dL)	0.2–1.2	6.2	6.2	2.1
INR	0.9–1.1	1.2	1.2	1.0
DAT – C3	Negative	Positive (3+)	Positive	Positive
DAT – IgG	Negative	Negative	Negative	Negative
Donath-Landsteiner Antibody	Negative	Positive	Positive	—
Cold Agglutinin Titer	Negative	Negative	Negative	—
Adenovirus PCR	Negative	Positive	—	—

On physical examination, the patient was tachycardic with scleral icterus and a mild systolic murmur. Abdominal examination demonstrated mild suprapubic tenderness without hepatosplenomegaly.

Medical, family, and social history

The patient had no prior medical conditions and was born full term with normal development. She lived with her father and three siblings. Family history was notable for maternal anemia of unclear etiology and gastrointestinal disorders in paternal relatives. Her father, a former Jehovah’s Witness, consented to blood transfusion if clinically necessary.

Differential diagnosis

Initial diagnostic considerations included CAD, PCH, warm AIHA, and cryoglobulinemia. A direct antiglobulin test (DAT) positive for complement (C3) but negative for IgG suggested a cold-reactive, complement-mediated process rather than warm AIHA [1,6].

Laboratory and diagnostic evaluation

Laboratory evaluation demonstrated findings consistent with severe hemolytic anemia, including decreased hemoglobin, reticulocytosis, hyperbilirubinemia, and elevated lactate dehydrogenase (LDH) (Table 1). Peripheral blood smear showed polychromasia and red blood cell agglutination without schistocytes. Expanded respiratory viral testing was positive for adenovirus, while hepatitis testing was negative. Blood bank studies revealed a complement-positive DAT, negative cold agglutinin titers, and a positive Donath-Landsteiner antibody, confirming the diagnosis of PCH. Abdominal ultrasound was unremarkable.

Management

The patient was managed with strict maintenance of normothermia, including pre-warming of intravenous fluids and avoidance of cold exposure. Immunomodulatory therapy included intravenous methylprednisolone and intravenous immunoglobulin (IVIG). Hematopoietic support with epoetin alfa and folic acid was provided to promote erythropoiesis. Empiric azithromycin was administered for possible *Mycoplasma pneumoniae* infection. A conservative transfusion strategy was employed, with transfusion reserved for symptomatic anemia or hemoglobin levels below 4.0 g/dL; transfusion was ultimately not required. Supportive care included antiemetics, analgesics, and close hemodynamic monitoring in the pediatric ICU.

## Discussion

PCH is a rare but important cause of acute hemolytic anemia in children and is characterized by biphasic IgG-mediated complement activation leading to intravascular hemolysis [2,4]. Unlike CAD, which is mediated by IgM antibodies and typically causes extravascular hemolysis, PCH is distinguished by intravascular red blood cell destruction and a DAT positive for complement C3 alone [1,5,6]. These diagnostic distinctions are critical, as management strategies and disease course differ substantially between AIHA subtypes.

Most pediatric cases of PCH occur following viral illnesses and follow a self-limited course with supportive management [2,3]. Adenovirus has been reported as a precipitating factor in only a small number of cases, underscoring the clinical relevance of this report [7]. While corticosteroids and IVIG are frequently used in clinical practice, evidence supporting their efficacy remains limited, and supportive care with normothermia and careful transfusion planning remains the cornerstone of therapy [6,9]. Recent reports of complement inhibition in refractory cases highlight evolving therapeutic strategies and further emphasize the central role of complement in PCH pathophysiology [9].

This patient’s favorable clinical course without transfusion, despite profound anemia, aligns with previously described pediatric cases and reinforces the importance of early recognition and appropriate diagnostic testing, including Donath-Landsteiner antibody assays, in suspected cases of cold-reactive hemolytic anemia.

## Conclusions

PCH is a rare but potentially severe form of pediatric AIHA that should be considered in children presenting with acute hemolysis following viral illness. This case highlights adenovirus as an uncommon but important trigger and underscores the diagnostic value of complement-positive DAT findings and Donath-Landsteiner antibody testing. Early recognition, maintenance of normothermia, supportive care, and individualized transfusion planning are essential to optimize outcomes and prevent complications in pediatric complement-mediated hemolytic anemia.
